# Patient and Family Perspectives on Integrated Transitional Care for Anorexia Nervosa in Mantova, Italy

**DOI:** 10.3390/nu17172830

**Published:** 2025-08-30

**Authors:** Debora Bussolotti, Giovanni Barillà, Antonia Di Genni, Martina Comini, Alberto Gallo, Mariateresa Torre, Laura Orlando, Beatrice Mastrolorenzo, Eva Corradini, Barbara Bazzoli, Francesco Bonfà, Andrea Mora, Luca Pasqualini, Elisa Mariantoni, Alessandro Cuomo, Despoina Koukouna, Paola Accorsi

**Affiliations:** 1 Department of Mental Health and Addictions, ASST Mantua, 46100 Mantua, Italy; 2 Department of Molecular Medicine, School of Medicine, University of Siena, 53100 Siena, Italy; 3 Department of Maternal and Child Health, ASST Mantua, 46100 Mantua, Italy

**Keywords:** anorexia nervosa, care transition, multidisciplinary team, client satisfaction, family-based treatment, adolescent psychiatry

## Abstract

Background/Objectives: The child and adolescent mental health service (CAMHS) hand-over to adult mental health service (AMHS) remains an ongoing shortfall in eating disorder (ED) treatment, typically in tandem with diagnostic drift, heightened suicide risk, and carer burn-out. We created one 14-to-25 Transition—ED track within our own unit, where a single multidisciplinary team continuously follows each patient and family across the CAMHS–AMHS boundary (via weekly joint paediatric and adult clinician meeting) without changing the individual psychotherapist, family therapist, or dietitian at the age 18 transition. We investigated the manner in which patients and parents perceive this model. Methods: A survey of two naturalistic parent cohorts—CAMHS (*n* = 16) and Transition—Adult arm (*n* = 15)—also joined, alongside the original group of young adults who had entered the programme during its set-up phase (*n* = 9). Here, the 14–25 pathway denotes one unified route of care across adolescence and young adulthood; the Transition—Adult arm is its ≥ 18-years component. All index patients had a primary DSM-5-TR diagnosis of restricting-type anorexia nervosa. Participants completed the Client Satisfaction Questionnaire-8 (CSQ-8; range 8–32) and four bespoke Continuity-of-Care items (1–4 Likert). Results: Overall, the caregivers in both cohorts were pleased (median CSQ-8 = 28.5 [CAMHS] vs. 27.0 [Transition]; *p* = 0.75). Continuity items were universally well rated across cohorts. Cohort parents reported a median of two unchanged core clinicians (i.e., the individual psychotherapist, the family therapist, or the dietitian), which was nonsignificantly positively correlated with CSQ-8 scores (ρ = 0.22). Early-group patients mirrored caregiver impressions (mean CSQ-8 = 27.0 ± 3.9). Conclusions: It is feasible and highly acceptable to both caregivers and anorexia nervosa young adults to have the same key staff and family-centred sessions over the 14-to-25 age span. Constrained by single-site study and small sample size, these preliminary data provide a rationale for wider implementation and controlled follow-up studies.

## 1. Introduction

Anorexia nervosa (AN) is a severe psychiatric illness [1] with onset most commonly in adolescence and prolonged clinical course often found. Despite peak early pubertal age of onset, prevalence remains equally elevated across young women’s ages. One third of cases will progress to severe and enduring presentation criteria (SE AN, >7 years) [2,3,4]. Lifetime prevalence is around 2–4% amongst females and around 0.3% amongst males [5]. In 2019, AN accounted for 153,058 disability-adjusted life years (DALYs) in the WHO Region of Europe, of which women accounted for 78% [6]; based on prolonged course, DALYs were attributable to individuals over 20 years of age by 117,946 (77%).

During the COVID-19 pandemic, referrals and presentations for eating disorders rose sharply in many countries. Services faced sustained pressure, with increased demand persisting beyond lockdown phases [7,8,9,10,11].

Malnutrition induced by AN produces diffuse medical and psychological morbidity, compromising growth, cognition, socio-emotional function and academic performance. Because it is a metabolically active organ, the brain is exquisitely sensitive: multicentre neuroimaging research documents a ~6% cortical volume loss in acute AN [12]. Neuropsychological outcomes are defective cognitive flexibility [13], memory defects [14] and abnormal social cognition [15]. Such neurocognitive alterations may persist after weight restoration, supporting the rationale for developmentally extended care [13,15].

A recent meta-analysis estimated the prevalence of deliberate self harm with and without suicidal intent to be 17% in AN [16], and AN carries the highest all-cause mortality of psychiatric illnesses [17].

Certain personality profiles and frequent mood or anxiety comorbidities also impair outcome [18]; for example, comorbid depression raises the odds of non-recovery sixfold after the age of 22 [2]. A long course of illness is also linked to cognitive, behaviour and neurobiological changes impairing responsiveness to treatment [19,20,21].

The costs are borne by carers too: families are subjected to prolonged emotional, logistical and financial stress along the illness course [22]. EU statistics report an average duration of untreated AN (DUED) of between 15 months and 2 years [20,23], with extended periods of latency between the onset of symptoms and diagnosis, assessment and evidence-based treatment [20,24]. Such inappropriate intervals are in part due to patient indecision; many are thus not offered disorder-specific care or face lengthy waits, and some are left entirely without treatment [23,25].

Even when treatment is sought, 20–30% of individuals with SE AN fail to respond to outpatient treatment and need more intense strategies such as inpatient or day patient treatment; of these, 30–40% need repeated admissions [18,26]. Timely and efficient access to specialist services is therefore essential to prevent a prolonged course and to optimise long term outcomes [27].

### 1.1. Beyond Body Mass Index: Diagnostic Migration and Family Burden

Weight restoration alone is rarely a marker of cure. Two meta-analyses involving more than 3000 patients confirmed that 30–60% of restricting type AN subjects switch within five years to binge–purge phenotypes or to bulimia nervosa (BN) [28,29,30]. Emergence of loss-of-control eating considerably increases shame and impulsivity and nearly triples suicidal behaviour risk [31,32,33]. Families often remain the primary caregivers for over a decade [34]. International guidelines today (American Psychiatric Association [APA] 2023; National Institute for Health and Care Excellence [NICE] guideline NG69, 2020) thus place the family in a central position, aided by a stable multidisciplinary team, in evidence-based adolescent and emerging adult care [27,35,36].

### 1.2. The Service Gap at the Age 18 Boundary

Despite this developmental rationalisation, psychiatric services in the majority of countries are nevertheless separated in administration between child and adolescent mental health services (CAMHS) and adult mental health services (AMHS). A background UK surveillance paper reported that fewer than 5% of adolescents experienced a smooth transfer and nearly half dropped out after CAMHS discharge [37]. Italian multicentre data report similar findings, with ongoing contact of only about 40% of cases six months after handover [23]. Divergent ideals of treatment (family-centred versus individual autonomous), unequal eligibility criteria and abrupt doctor changes are repeatedly reported deterrents [23]. Table 1 summarises crucial clinical and organisational shortcomings recognised by researchers and guidelines at child-to-adult hand-over for anorexia nervosa.

### 1.3. A 14-to-25 Integrated Service for Lombardy: Policy Framework and Local Implementation

To address these vulnerabilities, Lombardy Region Resolution DGR XI/7590 (15 December 2022) allocated dedicated funding for “transition” trajectories and mandated the implementation of integrated age 14–25 pathways [42]. In response, the Mantova Health Trust (ASST Mantova) developed Clinical Pathway 104 (PDTA 104), an operational protocol that merges paediatric and adult expertise into a single multidisciplinary Transition—ED team. Weekly case conferences involve paediatricians, child-and-adolescent and adult psychiatrists, internists, dietitians, educators, and individual and family psychotherapists. A key feature is relational continuity, with at least one core clinician—the individual psychotherapist, the family therapist, or the dietitian—remaining unchanged throughout the transition period. The pathway integrates Family-Based Treatment/New Maudsley principles—a model associated with roughly halved relapse and readmission within four years of remission [43,44]. This configuration aims to prevent handover fractures, sustain the therapeutic alliance, and uphold family-system support rather than a strict BMI-only focus.

### 1.4. Evidence Gap and Study Aims

Qualitative syntheses have emphasised timely planning, relational security and active family engagement in the process of transition [45], but quantitative evidence on caregiver- and patient-reported experience within integrated Italian services is equally lacking. For this cross-sectional study, four aims were therefore explored:

1. To establish local age of onset and of first diagnosis in the ED;
2. Comparing caregiver-reported satisfaction between two naturalistic cohorts: (a) parents of children under 18 years old treated within child and adolescent mental health services (CAMHS) and (b) parents of patients having completed ≥6 months in the Mantova Transition—ED programme;
3. To report on patient-reported satisfaction on a pilot subgroup of nine of the earliest enrolees to the transition pathway and examine it for a relationship with numbers of core clinicians retained beyond the age-18 boundary;
4. To assess whether caregiver satisfaction and perceived clinician continuity align with Mantova’s policy direction and national guidelines recommending integrated family-focused care from ages 14 to 25 [46,47].

## 2. Materials and Methods

### 2.1. Study Design and Setting

A single-site cross-sectional survey was undertaken in the Outpatient Eating Disorder Unit of ASST Mantova (Lombardy, Italy). The service offers an integrated “14-to-25” pathway outlined in PDTA 104—Disturbi della Nutrizione e dell’Alimentazione (Revision 1, 27 September 2024). The path outlined in local PDTA 104 (rev. 27 September 2024) offers continued multidisciplinary treatment for eating disorder (ED) service users for the age-range of mid adolescence to early adulthood. Participants were recruited consecutively during routine outpatient visits.

Caregivers and patients were approached from late June through 31 July 2025 during standard clinic appointments.

The study was approved by the Comitato Etico Territoriale (CET) Lombardia 4—ASST Cremona (protocol CET 69/25). All participants provided written informed consent after receiving a clear explanation of the study objectives and procedures and of their right to withdraw at any time. The investigation complied with the Declaration of Helsinki, Good Clinical Practice, and the EU General Data Protection Regulation (GDPR 2016/679). Further details are provided in the Institutional Review Board Statement.

Table 2 outlines the operational response of ASST Mantova and offers a summary of unifying elements of the 14-to-25 Transition—ED pathway outlined in PDTA 104.

### 2.2. Participants and Recruitment

#### 2.2.1. Caregiver Survey

Parents (or primary carers) were included if

1. They were accompanying a son or daughter to an outpatient consultation with an **initial diagnosis of restricting-type anorexia nervosa (AN-R)** according to Diagnostic and Statistical Manual of Mental Disorders, Fifth Edition, Text Revision (DSM-5-TR);
2. They had sufficient competence in Italian to respond to a brief computer-based questionnaire;
3. They provided written informed consent.

An independent research assistant, not involved in clinical practice, approached consecutive carers in the waiting area.

Two a priori cohorts were defined as follows:

(I) **CAMHS**: Parent of a patient < 18 years followed by the paediatric arm;
(II) **Transition—Adult arm**: Parent of a patient ≥ 18 years who, after ≥6 months in the same Transition—ED programme, had moved into its adult arm.

#### 2.2.2. Patient Pilot Survey

To capture experiences after meaningful exposure to the new service, the patient cohort comprised the first nine young adults with DSM-5-TR AN-R discussed by the multidisciplinary team within the first three months of the pathway’s start-up. This a priori, time-anchored definition avoided outcome-based selection and ensured that respondents had traversed key components of the CAMHS–AMHS interface.

Inclusion criteria were:

- Active treatment ≥6 months within the 14-to-25 Transition—ED pathway;
- First referral to local Adult Mental Health Service between January 2024 and March 2024 (programme start-up);
- Age 18–24 years at the time of the survey;
- Capacity to provide informed consent and complete questionnaires in Italian;
- Initial DSM-5-TR diagnosis of AN-R (as for the parent sample).

### 2.3. Survey Instruments

Service satisfaction was measured with the Client Satisfaction Questionnaire-8 (CSQ-8). The CSQ-8 comprises eight 4-point items [1,2,3,4]; the total score ranges within 8–32, with higher scores indicating greater satisfaction. The CSQ-8 is a widely validated satisfaction scale with robust psychometric properties across health-service settings; it shows high internal consistency and construct validity [47]. We used the standard Italian version already employed in routine clinical services.

To assess satisfaction with the dedicated 14–25 transition pathway and relational continuity across the age-18 boundary, caregivers—who are most directly involved in cross-age coordination—also completed four brief items (4-point Likert) developed for this service:

- C-Service: perceived impact of a dedicated 14–25 service (the transition model itself) on treatment effectiveness;
- C-Indiv: perceived benefit of keeping the same individual psychotherapist from 14 to 25;
- C-Family: perceived benefit of keeping the same family therapist from 14 to 25;
- C-Diet: perceived benefit of keeping the same dietitian from 14 to 25 *(Response anchors: 1 = strongly disagree; 4 = strongly agree)*.

In addition to the study scales, carers and patients reported patient sex, presenting age, parental ages, age at symptom onset, and age at first AN diagnosis. For carers in the Transition—Adult arm, we also recorded the number of unchanged clinicians (individual psychotherapist, family therapist, dietitian) on either side of the age-18 divide; these counts were used descriptively.

Surveys were administered immediately after a standard clinic appointment.

### 2.4. Procedure

Surveys were completed electronically on a clinic desktop computer using secure offline templates stored on the hospital network; responses were saved to anonymized, password protected study files and exported to a tabular dataset. All participants provided written informed consent prior to survey completion.

### 2.5. Statistical Analysis

The normality of continuous measures was explored using the Shapiro–Wilk test. As CSQ-8 sums and single items deviated slightly from normality (largest *p* = 0.057), non-parametric procedures were used overall.

- Caregiver comparisons (CAMHS vs. Transition—Adult arm):
  – Continuous/ordinal variables: Mann–Whitney U test;
  – Categorical variables: Fisher’s exact test.
- Estimation and effect sizes: Alongside *p*-values, we report effect sizes with 95% CIs—Cliff’s δ for Mann–Whitney tests (CIs via 5000-bootstrap resampling) and Spearman’s ρ (95% CI) for correlations.
- Multiplicity control: The four continuity items were tested in parallel; we therefore applied a Bonferroni-adjusted α = 0.0125 (0.05/4). All other tests used two-tailed α = 0.05.
- Exploratory correlations (e.g., within Transition—Adult caregivers) were used descriptively and were not included in confirmatory hypothesis testing owing to the small sample.
- Patient pilot cohort (*n* = 9): CSQ-8 was summarised descriptively only (mean ± SD; median; range), with no inferential comparison to caregivers given the convenience sample and the different respondent perspectives.
- Post hoc sensitivity: With *n*_1_ = 16 vs. *n*_2_ = 15 (two-sided α = 0.05; power 80%), the design would detect only very large between-group effects (approx. Cohen’s d ≈ 1.04, i.e., Cliff’s δ ≈ 0.54). Accordingly, non-significant results should not be interpreted as equivalence.

Software: Analyses were programmed in Python 3.11.

Abbreviations: CI, confidence interval; IQR, interquartile range; SD, standard deviation.

## 3. Results

### 3.1. Caregiver Sample

Thirty-one parents participated, 16 in child and adolescent mental health services (CAMHS) and 15 on the transition pathway—adult arm. Table 3 gives a summary of key demographics of their sons/daughters.

### 3.2. Parent-Reported Satisfaction and Continuity Perceptions

Overall satisfaction was high in both cohorts (CAMHS median 28.5; Transition—adult 27.0). Mann–Whitney U = 128.5, *p* = 0.750; Cliff’s δ = −0.071 (95% CI −0.488 to 0.333).

Continuity items were numerically higher in CAMHS, but none survived Bonferroni correction; effect sizes with 95% CIs are reported in Table 4.

### 3.3. Exploratory Analysis in Transition Caregivers

Transition—Adult caregivers reported a median of two stable core clinicians across the age-18 boundary (range 0–3). The correlation between number of stable core clinicians and CSQ-8 was small and non-significant (Spearman ρ = 0.22; *p* = 0.44).

### 3.4. Patient-Reported Satisfaction for Early Adopters

Nine early-adopter patients (eight female and one male), each with an initial restricting-type DSM-5-TR diagnosis of anorexia nervosa, met the inclusion criteria.

- Mean age: 18.9 ± 1.5 years.
- Mean illness duration: 4.0 ± 1.9 years.
- Retained clinicians per age boundary on average: 2.3 ± 0.9.

CSQ-8 showed mean 27.0 ± 3.9; median 27 (range 19–32). No inferential comparison with caregivers was planned.

## 4. Discussion

The transfer of a young person with an anorexia nervosa eating disorder from child services to adult services is regularly recognised as a “critical juncture” of course of treatment [21,22,24]. From an economic perspective, each unplanned Italian readmission for AN costs around EUR 12–15 k and is sustained at a smaller part of this figure by outpatient continuity [48]. Service models divide at precisely that point when the course of illness tends to become more complicated: restrictive control is regularly followed by cycles of behaviour and suicidality and shame [15,16,17]. Family-based continuity of the therapeutic connection has thus been recommended by each of the leading guidelines for adolescent and early-adult eating disorders [27,37,40,46]. The present survey provides for the first time quantitative evidence of our single-team, 14-to-25-year pathway for Mantova, meeting these ideals from the point of view of those most regular attenders at clinic: the patients’ parents. Early start-up patients reinforced this perception, having a mean CSQ-8 of 27/32, in exactly the same “high satisfaction” category as caregiver report ratings.

Satisfaction was very high in general (median CSQ-8~28/32) in each of the three cohorts, matching or surpassing benchmarks previously associated with robust therapeutic alliance and six-month retention [37,40]. Of particular note is that high drop-out rates—in common reaching 50% within a year of transfer—remain an ultimate flaw of age-segmented models [21,22]. That families of adult-pathway patients were equally positive in their ratings of the service compared to families of children still in child and adolescent mental health services (CAMHS) allays fears that the blurring of paediatric and adult expertise would dilute developmental nuance. Instead, data suggest a single team able to offer a more seamless and multidisciplinary service according to reported satisfaction.

Of special interest is also the continuum of the four “continuity” items. Most consistently rated most valuable by parents was the possibility of having the same clinician to work with and also having the same family therapist throughout adolescence and early adulthood—a fact consistent with qualitative accounts citing family work both as a unifying factor to maintain treatment on course and first to go when services become fragmented [24,39]. Though inter-group differences failed to reach the Bonferroni-adjusted significance level, the direction of effect is consistent with Mantova’s policy of retaining at least one familiar clinician in place (most often the family therapist or dietitian) beyond the age of eighteen years. From a developmental perspective, this is reasonable: executive functioning continues to mature into the mid-20s, which supports extended caregiver involvement [17,18,19,25].

The outcome also draws attention to a broader conceptual evolution: recovery is something beyond body-mass index. Continuity is valued by parents and by clients because it perpetuates attempts at intrusive thoughts about weight and shape, avoidance behaviours, and family communication repertoires long after normalised weight has occurred [10,11,12,28,45]. By placing dietitians, educators, individual and family psychotherapists, paediatricians, internists and psychiatrists in a weekly case conference setting, the Mantova model addresses illness in all its biopsychosocial complexity and not by specifying success in terms of kilogram endpoints. This connection is of clinical relevance: a meta-analysis of 47 reports reported a four-fold increase in suicidal thinking when patients moved on from restriction to binge–purge phenotypes [16].

Another factor to consider is that both young adult and adolescent variants of FBT are collaborative in practice, encourage flatmates/partners to attend sessions actively and are not strict about the eating autonomy of the patient [45]. Family therapists need to engage the broader relational network (partner, parents, flatmate, etc.) appropriately for the patient’s age.

### Strengths and Limitations

The present survey offers an early but informative window into the real-world impact of Lombardy’s 14-to-25 integrated pathway. Major strengths include (i) consecutive, naturalistic sampling during routine care: parents were recruited consecutively during routine outpatient consultations, with minimal selection bias; (ii) use of the well-validated CSQ-8 [47]; and (iii) clear a priori cohort definition, allowing for a direct comparison between the old child-service model and the new transition programme.

Several limitations temper generalisation. This study is underpowered (*n* = 31); with *n* = 16 vs. *n* = 15, only very large between-group effects would be detectable (see Section 2.5, post hoc sensitivity), and therefore non-significant differences should not be interpreted as equivalence. The cross-sectional design means that results may reflect local culture or clinician interest as much as the pathway itself, and it is not possible to infer causal links with long-term outcomes (relapse, hospitalisation, functional recovery). Most outcomes are self-reported by caregivers; while crucial to treatment adherence, parents may overestimate satisfaction or continuity if their child is now weight-restored. The four continuity questions, while face-valid and of direct relevance to policy objectives, lack formal psychometric validation; replication with standardised transition measures is warranted. The transition cohort by definition had longer illness duration and later diagnosis, which are confounders that could influence parental expectations independent of service design. Patient-reported results were available for only nine early start-up patients and were analysed descriptively, although their convergence with caregiver ratings supports the acceptability of the pathway.

Future research therefore must enhance sample size, track longitudinal clinical outcomes, and employ validated transition measures to ascertain whether the high satisfaction noted herein translates into tangible accomplishments in medical stability, psychological freedom and family functioning.

## 5. Conclusions

Too often, treatment for eating disorders loses momentum at the precise moment care shifts from paediatric to adult services—just when symptoms may evolve, suicide risk peaks, and families are exhausted. The Mantova PDTA 104 pathway offers a pragmatic alternative: one multidisciplinary team that follows each patient from mid-adolescence into young adulthood, ensuring at least one familiar clinician and ongoing family involvement throughout. Our data show that this integrated model is not only workable but genuinely appreciated: caregivers and the inaugural group of 14-to 25-year-olds reported similarly high Client Satisfaction Questionnaire-8 scores. Such across-the-board endorsement indicates that blending paediatric and adult expertise can preserve, and perhaps enhance, the therapeutic alliance. Although preliminary, these findings support further investment in unified 14-to-25 services and underscore the need for larger, longitudinal studies to examine effects on relapse, diagnostic crossover, functional recovery, and cost-effectiveness. From a service-design perspective, a practical “minimum viable” configuration emerges from our data: a single cross-age multidisciplinary team; continuity of named clinicians for the individual psychotherapist, the family therapist, and the dietitian across the age-18 boundary; joint CAMHS–AMHS case reviews with shared care plans; and a staged hand-over rather than a one-off transfer. Adopting the Mantova blueprint more widely could flatten both the clinical relapse curve and the economic one, reminding us that recovering from anorexia nervosa is a long, family-centred journey, not a dash to a target body-mass index.

## Figures and Tables

**Table 1:** Key challenges at the child-to-adult transition in anorexia nervosa care.

Domain	Specific Critical Issue	Typical Consequence
**Early detection and referral**	Diagnostic **drift unrecognised** (AN-R → binge/purge forms) [28,29]	Later presentation to adult team with entrenched behaviours
	**BMI-only focus** after weight restoration [38]	Cognitive symptoms underestimated → false “remission”
**Service organisation**	**Age-18 “cliff edge”** between CAMHS and AMHS [37]	Up to 50% disengagement within 6 months
	Divergent treatment cultures (family-centred vs. autonomous) [27]	Abrupt loss of family sessions, rupture of therapeutic alliance
**Family involvement**	**Reluctance to “start over”** with new adult team [34]	Reduced parental monitoring just as binge–purge risk rises
	Caregiver burn-out after crisis phase [36]	Drop-out during maintenance → higher relapse and self-harm
**Multidisciplinary continuity**	**Discontinuity** of physicians, dietitian and individual/familiar psychotherapist [39]	Drop-out during transition; stalled cognitive re-processing
	Fragmented medical–psychiatric records [40]	Delayed detection of osteopenia, cardiac sequelae
**Risk management**	**Surge in impulsivity** when binge–purge emerges [32]	Three-fold rise in suicidal ideation/attempts
	**Lack of shared crisis protocols** across services [41]	Unplanned emergency admissions, higher costs

**Table 2:** Mantova 14–25 Transition—ED Model (PDTA 104, Rev. 2024).

Component	Operational Detail (ASST Mantova)	Intended Clinical Impact
Unified MDT	*Weekly joint conference* with paediatricians, neuro-psychiatrists, adult psychiatrists, internists, dietitians, educators, individual & family therapists	Eliminates hand-over fracture; aligns medical & psychosocial plans
Relational continuity	≥1 constant clinician (psychologist/family therapist/dietitian) accompanies patient across age 18, from CAMHS to Adult services	Preserves therapeutic alliance; buffers stigma & shame
Family-centred care	FBT-informed sessions continue into young adulthood; parents receive psycho-education and relapse-prevention coaching	Sustains meal supervision & symptom monitoring when autonomy rises
Development-tailored intensity	Case intensity adjusted to developmental milestones (exam periods, university entry, work) rather than chronological age alone	Prevents drop-out at life transitions; supports functional recovery

Abbreviations: MDT = multidisciplinary team; FBT = family-based treatment.

**Table 3:** Index-patient characteristics reported by caregivers.

Variable	CAMHS (*n* = 16) Median [IQR]	Transition—Adult Arm *(n* = 15) Median [IQR]	*p*-Value ^†^
Age (years)	15.0 [13.0–16.0]	19.0 [18.0–20.5]	<0.001
Age at symptom onset (years)	13.0 [12.0–14.3]	15.0 [14.0–16.0]	0.096
Age at first ED diagnosis (years)	14.0 [12.0–14.3]	16.0 [15.0–17.0]	0.017

*All probands carried an initial DSM-5-TR diagnosis of restricting-type anorexia nervosa*. ^†^ Mann–Whitney U test.

**Table 4:** Caregiver outcomes: CSQ-8 and continuity items.

Outcome (Likert 1–4 Unless Stated)	CAMHS—Median [IQR]	Transition—Adult Median [IQR]	*p*-Value ^†^	Cliff’s δ (95% CI)
CSQ-8 total (8–32)	**28.5 [26.0–30.8]**	**27.0 [24.0–30.0]**	**0.750**	**−0.071 (−0.488 to 0.333)**
Dedicated 14–25 service helpful (C-Service/Q9)	4.0 [3, 4]	3.0 [2, 4]	0.192	−0.242 (−0.568 to 0.104)
Same individual psychotherapist (C-Indiv/Q10)	4.0 [3, 4]	3.0 [2, 4]	0.330	−0.183 (−0.529 to 0.171)
Same family therapist (C-Family/Q11)	3.5 [3, 4]	3.0 [3, 3]	0.067	−0.333 (−0.633 to 0.008)
Same dietitian (C-Diet/Q12)	4.0 [3, 4]	3.0 [2, 4]	0.128	−0.292 (−0.642 to 0.104)

^†^ Mann–Whitney U test; Bonferroni-adjusted α = 0.0125 for continuity items. Effect sizes are Cliff’s δ with 95% bootstrap CIs (5000 resamples).

## Data Availability

The data presented in this study are available on reasonable request from the corresponding author. The data are not publicly available due to privacy restrictions.
